# Femur, Tibia, and Fibula Fractures Secondary to Youth Soccer: A Descriptive Study and Review of the Literature

**DOI:** 10.7759/cureus.8185

**Published:** 2020-05-18

**Authors:** Peter Zaki, Sayyar Khakimov, Joseph Hess, William Hennrikus

**Affiliations:** 1 Orthopaedic Surgery, Penn State College of Medicine, Hershey, USA; 2 Radiation Oncology, University of Washington, Seattle, USA; 3 Pediatric Surgery, Penn State Health Milton S. Hershey Medical Center, Hershey, USA; 4 Orthopaedic Surgery, Penn State Health Milton S. Hershey Medical Center, Hershey, USA

**Keywords:** injuries, lower extremity, sport, football, pediatric

## Abstract

Objectives

Soccer is the most popular sport in the world and is one of the top sports with increased participation. Despite the vast and increasing numbers of soccer players, limited data are available on pediatric lower extremity injuries. In particular, the purpose of the study is to describe the epidemiology of femur, tibia, and fibula fractures secondary to youth soccer.

Methods

A retrospective review concerning soccer-related femur, tibia, and fibula fractures was conducted in children under the age of 18 years from January 1, 2000 to December 31, 2015 with statewide data from the Pennsylvania Trauma Systems Foundation (PTSF), Mechanicsburg, PA.

Results

A total of 258 youth soccer players were admitted for femur, tibia, and fibula fractures from 2000 to 2015. These fractures constituted 33% of soccer-related injuries in youth admitted at trauma centers. Sixty-five percent of the fractures involved the tibia and 34% involved the femur. Body contact injury resulted in 54% of the fractures and non-body contact injury resulted in 46% of the fractures. Athletes the age of 13 and older sustained 67% of the fractures and were more likely to incur contact injuries (p-value=0.000041) than those less than 13. Males sustained 67% of the fractures, and gender was not associated with the mechanism of injury (p-value=0.43). Open fractures included 10% of tibia fractures and did not occur in femur fractures. The growth plate was involved in 24% of the femur fractures and 17% of the tibia fractures.

Conclusion

Youth soccer has the potential for serious femur, tibia, and fibula fractures. Intervention programs should aim at reducing non-body contact mechanism in children < 13 years of age and body contact mechanism in children ≥ 13 years of age. Further research should investigate injury prevention methods such as potentially reducing body contact mechanism by improving the effectiveness of shin guards.

## Introduction

Soccer is the most popular sport in the world [1]. The Fédération Internationale de Football Association (FIFA) estimates that there are 270 million players in 207 countries with most registered players being under the age of 18 years [1]. The United States (US) soccer youth association reported 3,020,442 registered players in 2000 and 3,055,148 in 2015. With this growing participation, the prevalence of soccer-related injuries presenting to pediatricians is likely to rise [2]. In fact, Smith, Chounthirath, and Xiang found that the annual incidence of youth soccer injuries more than doubled between 1990 and 2014 in their analysis of the National Electronic Injury Surveillance System [3].

While lower extremity injuries are the most common injuries in soccer, limited data are available on these injuries in the pediatric population [4-9]. Furthermore, fractures of the femur, tibia, and fibula are hallmarked by delayed return to sport and complications [10]. Despite the severity of these injuries, their incidence, mechanisms, treatment, prognosis, and prevention have been understudied [10]. Moreover, the likely mechanism of injury in soccer has been inconsistent in literature [11-18]. The purpose of this study is to address the knowledge gap on femur, tibia, and fibula fractures in youth soccer, including the incidence, mechanisms, treatment, and prognosis of these injuries. This study investigates the epidemiology of youth soccer-related femur, tibia, and fibula fractures treated in all accredited trauma centers in the state of Pennsylvania and identifies areas for potential preventative intervention. The authors hypothesize that femur, tibia, and fibula fractures in youth soccer are major in severity, and that tibia fractures are more common than femur and fibula fractures due to body contact mechanism.

## Materials and methods

A retrospective review about soccer-related femur, tibia, and fibula fractures was conducted in children under the age of 18 years from January 1, 2000 to December 31, 2015 with statewide data from the Pennsylvania Trauma Systems Foundation (PTSF), Mechanicsburg, PA. Since 1984, the PTSF has been the accrediting body for the Commonwealth of Pennsylvania trauma programs, which includes hospitals and medical centers. There are currently 43 trauma centers. The database has been used to publish previous studies; a PubMed advanced builder search using “Pennsylvania trauma systems foundation” in “Title/Abstract” field yielded 30 results on May 9, 2019.

The database provided a wide array of information in an excel file compiling data inputted by each trauma center. Trauma centers include patients into the database if they are admitted for trauma-related injuries. The dataset has more than 300 data elements (e.g. institution and trauma numbers, mechanism of injury as described by E-codes, injury severity score (ISS), text describing the injury, text describing cause of injury, and number of hospital days). E-codes are used by the International Classification of Diseases (ICD) to describe external causes of morbidity. ISS is an established medical score that assesses trauma severity and correlated with mortality, morbidity, and hospitalization time after trauma. There were 83,286 patient records in the data provided to the investigators. Soccer injuries were filtered using the equation =IF(ISERROR(SEARCH(“soccer”, BE2,1)), “”,“1”)). A similar equation was then applied to filter for femur, tibia, and fibula fractures. Further filtering for data analysis was performed using the filter drop down menu. Inclusion criteria included males and females with an age between 0 and 18 years with a soccer-related femur, tibia, or fibula fracture. There were no exclusion criteria.

The incidence of injuries was calculated per youth soccer players in the state of Pennsylvania per year, which was estimated based on US soccer youth association reports of players and the state-national population ratio in 2000 and 2015 per the US census bureau. Data analyzed included age, gender, mechanism of injury, hospital stay (in days), ISS, location of injury, type of injury, day of the week, and date of injury.

The severity of an injury was classified based on the duration of absence from play. The four categories were minor (2-3 days), mild (4-7 days), moderate (1-4 weeks), and major (>4 weeks) [8]. There are several studies showing the majority of acute injuries in soccer to be due to trauma [4,7,11-13]. Player-to-player contact mechanisms have been reported to cause injuries more commonly than non-body contact mechanisms, but this was not consistent in other studies [4,7,14-18]. Therefore, for the current study on femur, tibia, and fibula fractures, the mechanism was dichotomized between (player-to-player) body contact and non-body contact. Intentional or accidental striking against another player was classified as body contact injuries. Injuries that occurred while shooting, running, twisting/turning, and landing were classified as non-body contact injuries. Although traumatic, injuries from colliding into goal posts or the ground, whether from trip/push or shoe-surface interaction were classified as non-body contact injuries [4,16-18]. Injuries were stratified categorically by gender, age, mechanism of injury, bone injured, location of the bone injured, and ISS. Bone injured was femur, tibia, or fibula. Location of bone injured was proximal, midshaft, distal (which included the malleoli), or growth plate. The categories were then analyzed for associations using chi-square tests. Statistical significance of alpha level was determined using a priori criteria p < 0.05. IBM Statistical Package for the Social Sciences (SPSS) version 25.0 (IBM Corp., Armonk, NY) was used to conduct all statistical analyses. This study was approved by the College of Medicine Institutional Review Board.

## Results

Demographics and player data

**Figure 1:**
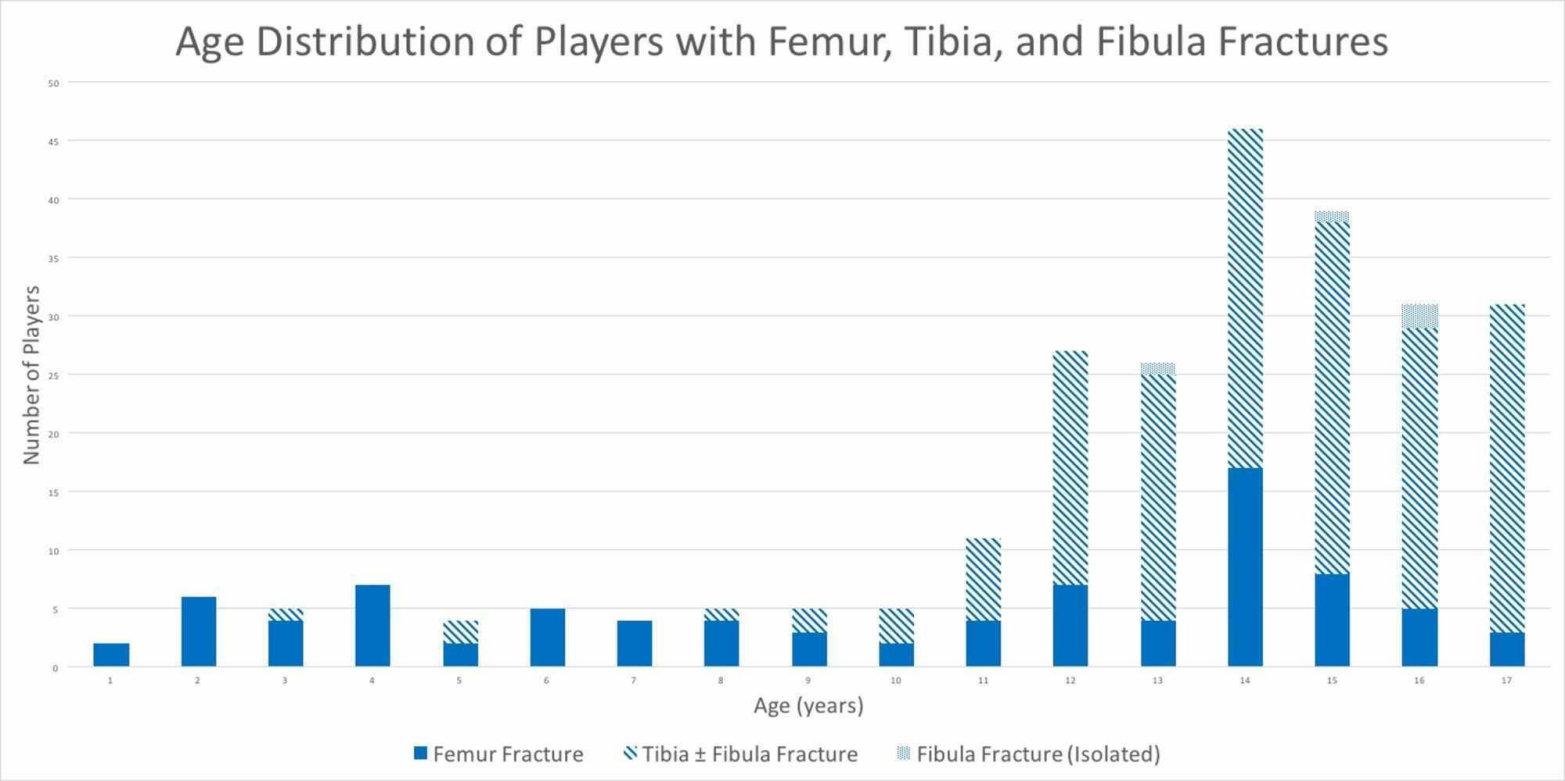
Age distribution of players with femur, tibia, and fibula fractures Femur fractures were more common in players < 13 years of age, while tibia and fibula fractures were more common in players ≥ 13 years of age.

Femur, tibia, and fibula fractures constituted 33% of soccer-related injuries in youth admitted at trauma centers. A total of 258 youth soccer players were admitted for femur, tibia, or fibula fractures from 2000 to 2015 at 27 different trauma centers in the database. The estimated incidence of admitted femur, tibia, and fibula fractures per number of soccer players per year increased from 0.0062% in 2000 to 0.021% in 2015. The average age was 13. Sixty-seven percent of the patients were 13 years of age or older and 33% of patients were less than 13 years of age (Figure 1). Musculoskeletal genetic diseases was identified in three patients with femur fractures and none in tibia fractures. These patients included a girl with osteogenesis imperfecta type I and a boy and girl with fibrous dysplasia; all had their fractures before the age of 12.

Injury data

**Figure 2:**
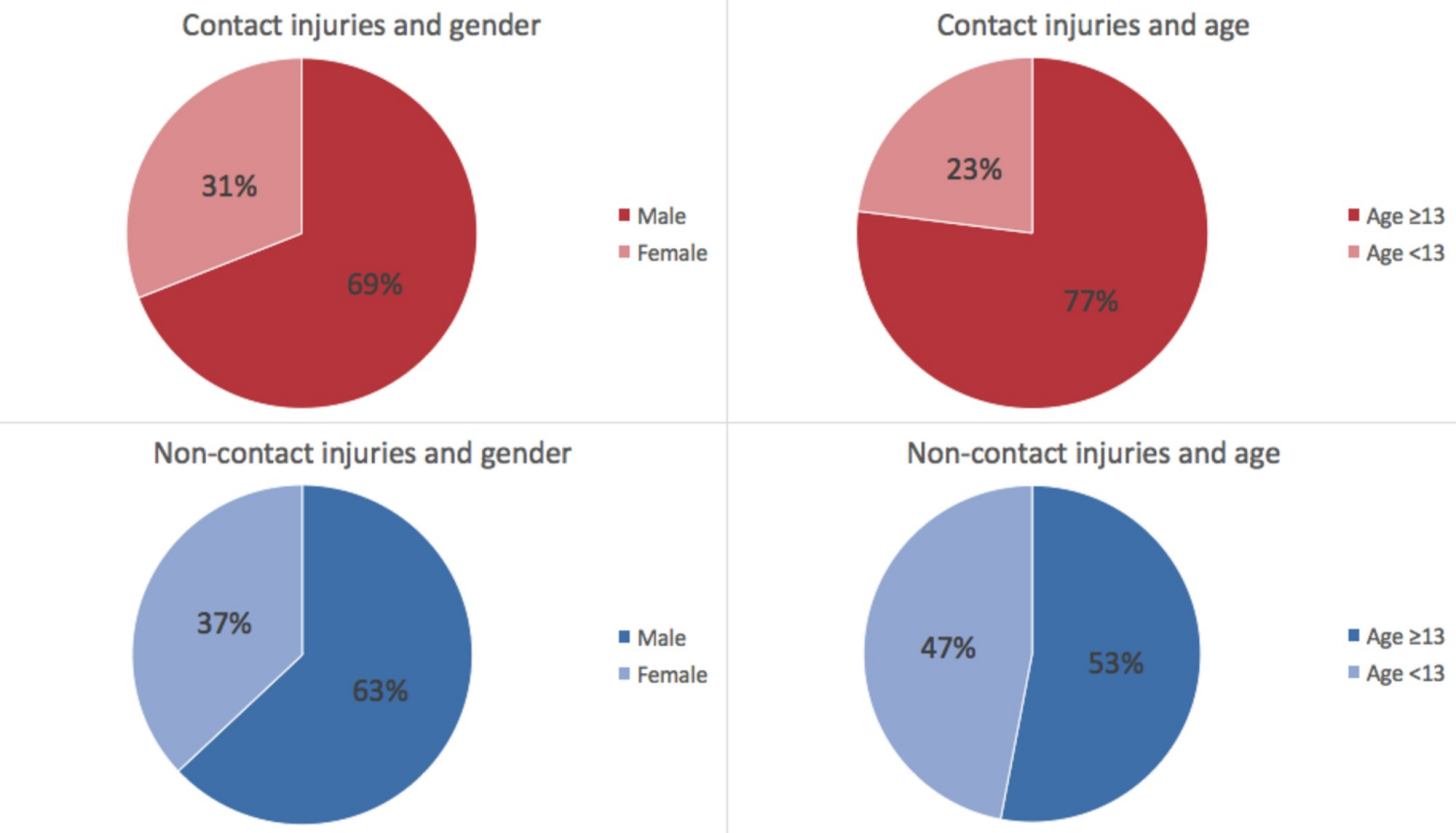
Comparison of mechanism of femur, tibia, and fibula fractures between males and female and between ages <13 years old and ≥ 13 years old Males and females were found to have an insignificant association with mechanism of injury (p-value = 0.43). Players <13 years of age were significantly more likely to have non-contact mechanism injuries, while players ≥ 13 years of age were more likely to have contact mechanism injuries (p-value = 0.000041).

Non-body contact resulted in 120 (46%) fractures and contact resulted in 138 (54%) fractures. Patients < 13 years of age were significantly more likely to be subjected to non-body contact mechanism fractures while those ≥ 13 years of age were significantly more likely to suffer body contact mechanism fractures (p-value = 0.000041) (Figure 2).

Tibia and fibula fracture data

A total of 168 tibia fractures were identified and patients who were ≥ 13 years of age sustained 79% of these fractures. Players ≥ 13 years (p-value = 0.10) and males (p-value = 0.95) were more likely than players < 13 years and females to suffer tibia injuries due to body contact mechanisms, but the difference was not statistically significant (Table 1). The tibia was fractured more often than femur and fibula. Most tibia fractures occurred due to body contact mechanisms. The shaft of the tibia was most commonly fractured (67%) for both body contact and non-body contact injuries. Body contact mechanisms caused significantly more shaft fractures (p-value < 0.0031) (Table 1). Of all tibia fractures, 72% had a concomitant fibula fracture. Of the paired tibia and fibula fractures 13% were open. Of the open fractures, 69% were due to body contact mechanisms, 75% occurred in males, and 56% occurred in those ≥ 13 years old. Neither age nor gender demonstrated significant association with open fractures or the location of bone fractures (Table 2). The growth plate was involved in 17% of fractures. Of all tibia growth plate fractures, 59% occurred due to non-body contact mechanisms, 66% occurred in males, and 76% occurred in those ≥ 13 years old. Finally, there were four isolated fibular fractures, one of which involved the growth plate. The average ISS of tibia fractures was 3.8 ± 2.1. Of the eight patients with tibial fractures and available follow-up data, 75% required surgery and 50% had post-op complications or required repeat surgery. The average time to return to play was 8.3 ± 2.6 months.

**Table 1:** Comparison of injury mechanisms of tibia fractures between males and females, players <13 years old and ≥ 13 years old, and location of bone (proximal, shaft, and distal) Males and females were found to have an insignificant association with mechanism of injury (p-value = 0.95). Players <13 years of age and ≥ 13 years of age were found to have an insignificant association with mechanism of injury (p-value = 0.10). Shaft tibia fractures were significantly more likely to occur due to contact mechanisms, while proximal and distal fractures were more likely to be due to non-contact mechanisms (p-value =0.0031).

Tibia Fractures								
	Male	Female	Age <13	Age ≥13	Proximal	Shaft	Distal	Total
Total	116 (69%)	52 (31%)	36 (21%)	132 (79%)	14 (8%)	113 (67%)	41 (25%)	168 (100%)
Contact	72 (69%)	32 (31%)	18 (17%)	86 (83%)	6 (6%)	80 (77%)	18 (17%)	104 (100%)
Non-Contact	44 (69%)	20 (31%)	18 (28%)	46 (72%)	8 (13%)	33 (52%)	23 (35%)	64 (100%)

**Table 2:** Comparison of location of tibia fractures between males and females and players <13 years old and ≥ 13 years old Males and females were found to have an insignificant association with location of bone fracture (p-value = 0.33). Players <13 years of age and players ≥ 13 years of age were found to have an insignificant association with location of bone fracture (p-value = 0.17)

Tibia Fractures					
	Male	Female	Age <13	Age ≥13	Total
Proximal	8 (57%)	6 (43%)	3 (21%)	11 (79%)	14 (100%)
Shaft	82 (73%)	31 (27%)	20 (18%)	93 (82%)	113 (100%)
Distal	26 (63%)	15 (37%)	13 (32%)	28 (68%)	41 (100%)

Femur fracture data

A total of 87 femur fractures were identified and patients who were < 13 years of age sustained 57% of the femur fractures. Players < 13 years of age were significantly more likely to have non-body contact mechanism fractures whereas those that were ≥ 13 years of age were significantly more likely to have body contact mechanism fractures (p-value = 0.0054) (Table 3). Males were more likely to suffer femur injuries due to body contact mechanisms but the difference was not statistically significant (p = 0.22) (Table 3). The shaft (44%) and distal (44%) femur were more commonly fractured than the proximal femur (12%). Distal femur fractures were significantly more likely to occur due to body contact mechanisms, while proximal and shaft injuries were more likely to occur from non-body contact mechanisms (p-value = 0.0039) (Table 3). Similarly, players ≥ 13 years old were significantly more likely to have distal femur fractures, whereas those < 13 years old were more likely to have shaft and proximal femur fractures (p-value = 1.5e-7) (Table 4). There were no open femur fractures. The growth plate was involved in 24% of fractures. Of all femur growth plate fractures, 67% occurred due to non-body contact mechanisms, 52% occurred in males, and 71% occurred in those ≥ 13 years old. One youth soccer athlete sustained a distal femur fracture and concomitant tibia fracture. The average ISS of femur fractures was 3.8 ± 1.9. Of the six patients with femur fractures and available follow-up data, 67% required surgery and 50% had post-op complications or required repeat surgery. Two patients (33%) had physeal arrest in the injured femur, one had a bilateral epiphysiodesis while the other experienced overgrowth phenomenon in the ipsilateral tibia. The average time to return to play was 9.5 ± 2.7 months.

**Table 3:** Comparison of injury mechanisms of femur fractures between males and females, players <13 years old and ≥ 13 years old, and location of bone (proximal, shaft, and distal) Males and females were found to have an insignificant association with mechanism of injury (p-value = 0.22). Players <13 years of age were significantly more likely to have non-contact mechanism injuries, while players ≥ 13 years of age were more likely to have contact mechanism injuries (p-value = 0.0054). Distal femur fractures were significantly more likely to occur due to contact mechanisms, while proximal and shaft femur injuries were more likely to be due to non-contact mechanisms (p-value =0.0039). One femur shaft fracture in a male teenager occurred due to an unknown mechanism.

Femur Fractures								
	Male	Female	Age <13	Age ≥13	Proximal	Shaft	Distal	Total
Total	54 (62%)	33 (38%)	50 (57%)	37 (43%)	11 (12%)	38 (44%)	38 (44%)	87 (100%)
Contact	23 (70%)	10 (30%)	13 (39%)	20 (61%)	2 (6%)	9 (27%)	22 (67%)	33 (100%)
Non-Contact	30 (57%)	23 (43%)	37 (70%)	16 (30%)	9 (17%)	28 (53%)	16 (30%)	53 (100%)

**Table 4:** Comparison of location of femur fractures between males and females and players <13 years old and ≥ 13 years old Males and females were found to have an insignificant association with location of bone fracture (p-value = 0.77). Players <13 years of age were significantly more likely to have proximal and shaft femur fractures, while players ≥ 13 years of age were more likely to have distal femur fractures (p-value = 1.5e-7).

Femur Fractures					
	Male	Female	Age <13	Age ≥13	Total
Proximal	7 (64%)	4 (36%)	8 (72%)	3 (28%)	11 (100%)
Shaft	22 (58%)	16 (42%)	33 (87%)	5 (13%)	38 (100%)
Distal	25 (66%)	13 (34%)	9 (24%)	29 (76%)	38 (100%)

## Discussion

Age group was associated with mechanism of injury and location of fracture

Analysis of femur, tibia, and fibula fractures by age revealed that patients < 13 years of age were significantly more likely to suffer non-body contact mechanism fractures while those ≥ 13 years of age were significantly more likely to suffer body contact mechanism fractures. An increase in player-to-player contact mechanism with age is consistent with a finding in a study of overall youth soccer-related injuries [19]. In addition, the incidence of injuries has been reported to increase with age in youth soccer and even surpass the incidence of injuries in adult soccer at the age of 17-18 [12]. The association between age and injury suggests that age could potentially be used to predict the cause of youth soccer-related femur, tibia, and fibula fractures. Furthermore, literature shows age-specific prevention programs, such as “11+” for those greater than 13 years of age and “11+ Kids” for those 7 to 13 years of age, have demonstrated a reduction in youth soccer injuries overall [5,13,20].

This study found age group to not only be associated with the mechanism of injury, but also associated with the location of the fracture. Femur fractures were more prevalent in those < 13 years of age, while tibia fractures occurred more often in those ≥ 13 years of age. Fractures of the tibia occurred more commonly than femur and fibula fractures. Most tibia fractures occurred in the shaft and were due to body contact mechanisms. This suggests that our hypothesis was true - tibia fractures were more common than femur fractures due to body contact mechanism (e.g. shin-to-shin).

Body contact mechanism caused the majority of injuries

This study associated most fractures with body contact mechanisms. This is consistent with studies on overall injuries in soccer, which is classified as a high- to moderate-intensity contact sport [21]. Most injuries occur from player-to-player contact [4,7,11,15]. However, other studies found that non-body contact mechanisms caused the majority of injuries [16-18]. Several studies also support the widely held belief that the injury rate is higher during competition than training [2,9,12,22]. This may be because as the competition increases, players tend to have more body contact (i.e. sliding and tackling). The authors were unable to determine whether injury rates increased during competition since that information was not available for the majority of patients. However, more fractures occurred on weekends (54 fractures on Saturday, 42 on Fridays, and 41 on Sundays), which is when tournaments occur [22]. Trauma centers should expect and prepare for these injuries to happen more often on the weekends. While it may be impractical to decrease competition in soccer, stricter adherence to rules and improved education on safe tackling and shooting should be considered.

Foul-play has been associated with a significant number of contact-related injuries [2,11,23]. One study of youth athletes in nine different sports across 100 US high schools identified injuries due to illegal activity, and found that girls’ soccer (11.9%) and boys’ soccer (11.4%) had the second and third highest rates of such injuries, only preceded by girls’ basketball (14%) [24]. Officials can emphasize safe play with respect for one’s opponents, thereby playing significant roles in reducing body contact injuries [2,25]. Interventions such as closer adherence to the rules and using better equipment have been proposed to reduce the number of injuries [26]. FIFA made shin guards a required basic equipment for players in 1990 and a study from 1990 to 1994 showed a decrease in incidence of soccer-related tibia fractures compared to a study from 1988 to 1990 [27,28]. However, wearing a shin guard does not eliminate injuries. Two studies found that the majority (90% and 96%) of lower leg fractures occurred while a player was wearing shin guards [10,29]. Previous studies and the current findings suggest that further research on preventing femur, tibia, and fibula fractures could focus on improving the effectiveness of shin guards in order to reduce body contact injuries.

Youth soccer injuries represented a cost for players and the health care system

**Figure 3:**
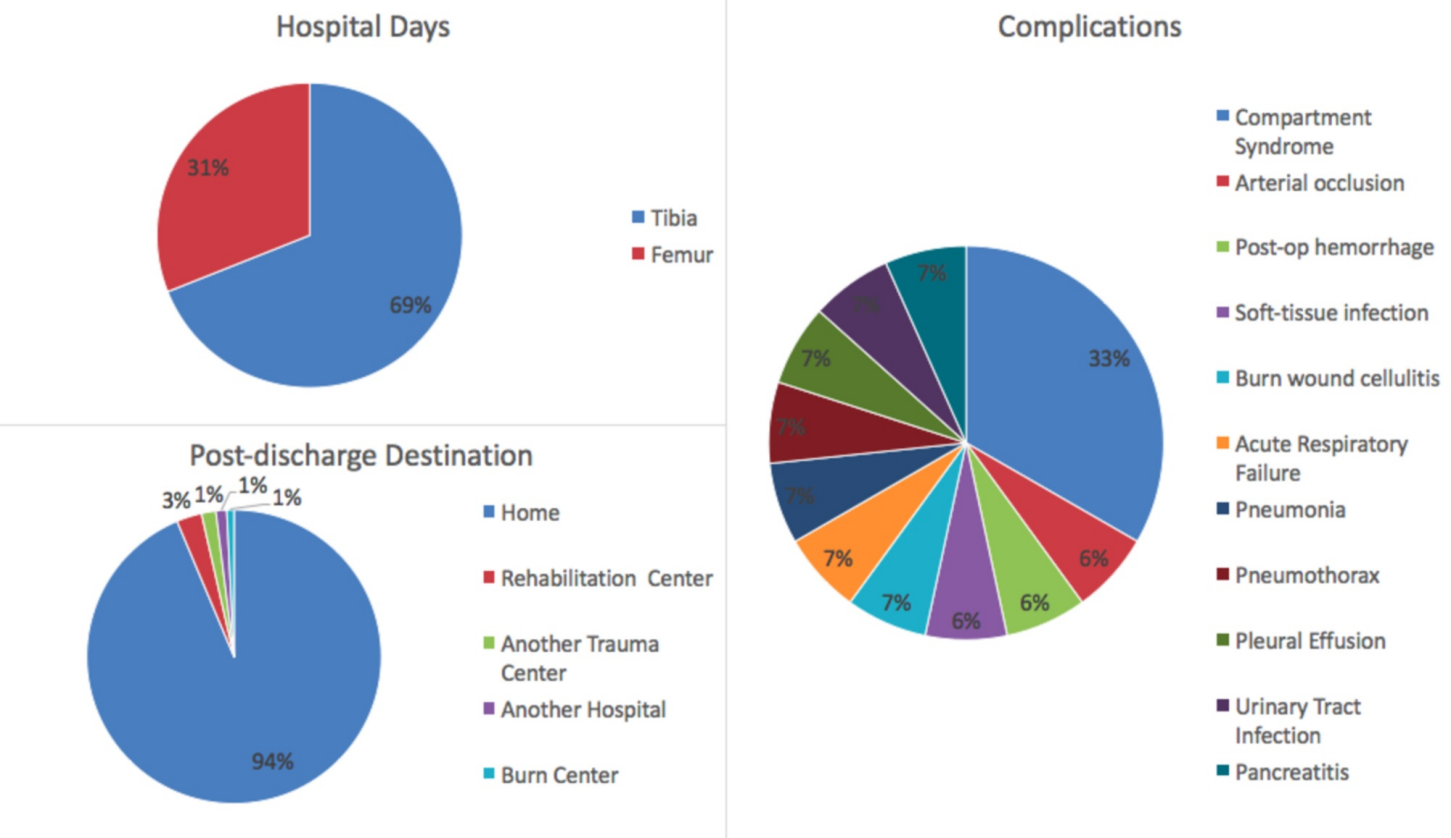
Hospital course and post-discharge destination for femur and tibia fractures Patients with tibia fractures had a total of 649 hospital days, median of two days, and range of 1-51 days. Patients with femur fractures had a total of 292 hospital days, median of two days, and range of 1-34 days. The 51-day course was complicated by an acute arterial occlusion and the 34-day course was complicated by pancreatitis. There were a total of 15 complications that occurred during the patients’ hospital stays. Compartment syndrome was the most common complication and only occurred in patients with tibia fractures. The most common post-discharge destination was home.

Youth soccer players tend to have a higher relative injury risk and greater prevalence of injuries compared to older players, and thus are a major driver of soccer-related healthcare costs [9,22,23,29]. The incidence of femur, tibia, and fibula fracture per youth soccer players per year increased from 0.0062% to 0.02% during the 15 year study period. Despite an incidence rate of less than one percent, femur, tibia, and fibular fractures constituted 33% of soccer-related injuries in youth admitted at trauma centers. This suggests these fractures are severe in nature, and our hypothesis was true in that these fractures are major in severity because the average time to return to play was around nine months. These consequences could lead to prolonged recovery time or missed recruiting opportunities. Costs further amount from hospital admissions, potential complications, and rehabilitation (Figure 3) [10]. For a subset of patients with available follow-up data, the majority required surgery, and about half of them had post-op complications or required repeat surgery. About one-third of these patients with femur fractures suffered from physeal arrests. In addition to hindering growth, an injury can also increase the risk of future injuries [9,30]. The rising incidence and possibility of such potential outcomes underscore the importance of developing prevention programs for youth soccer-related femur, tibia, and fibula fractures.

Limitations

Limitations of this study include the ability to assess injury risk due to the retrospective nature of the study as well as limited exposure data. Information including but not limited to the setting (i.e. game or practice) of the injury, weather conditions, shoe type, field surface, player skill level, and position played were unavailable. Another limitation is the lack of generalizability due to data being gathered from multiple institutions in one state. The number of fractures reported may be an underestimate as some fractures such as stress, malleolar, or condylar may have presented to outpatient clinics or emergency departments and not led to trauma activation at accredited trauma centers. Additionally, there is no information on the associated economic burden of these fractures. This study establishes the foundation for further investigation. Further studies may focus on exposure data and potential interventions.

## Conclusions

This is the first study to focus on femur, tibia, and fibula fractures due to youth soccer. These findings suggest that youth soccer has the potential for serious femur, tibia, and fibula fractures. Intervention programs should be aimed at reducing non-body contact mechanism in children < 13 years of age and body contact mechanism in children ≥ 13 years of age. Further research should investigate injury prevention methods such as potentially reducing body contact mechanism by improving the effectiveness of shin guards.

## References

[1] Stamm H, Lamprecht M (2020). FIFA big count 2006: 270 million people active in football. FIFA.

[2] Koutures CG, Gregory AJM (2010). Injuries in youth soccer. Pediatrics.

[3] Smith NA, Chounthirath T, Xiang H (2016). Soccer-related injuries treated in emergency departments: 1990-2014. Pediatrics.

[4] Faude O, Rößler R, Junge A (2013). Football injuries in children and adolescent players: are there clues for prevention?. Sports Med.

[5] Rössler R, Junge A, Bizzini M (2018). A multinational cluster randomised controlled trial to assess the efficacy of ’11+ kids’: a warm-up programme to prevent injuries in children’s football. Sports Med.

[6] Inklaar H (1994). Soccer Injuries. Sports Med.

[7] Kakavelakis KN, Vlazakis S, Vlahakis I, Charissis G (2003). Soccer injuries in childhood. Scand J Med Sci Sports.

[8] Price RJ, Hawkins RD, Hulse MA, Hodson A (2004). The Football Association medical research programme: an audit of injuries in academy youth football. Br J Sports Med.

[9] Wong P, Hong Y (2005). Soccer injury in the lower extremities. Br J Sports Med.

[10] Boden BP, Lohnes JH, Nunley JA, Garrett WE Jr (1999). Tibia and fibula fractures in soccer players. Knee Surg Sports Traumatol Arthrosc.

[11] Emery CA, Meeuwisse WH, Hartmann SE (2005). Evaluation of risk factors for injury in adolescent soccer: implementation and validation of an injury surveillance system. Am J Sports Med.

[12] Söderman K, Adolphson J, Lorentzon R, Alfredson H (2001). Injuries in adolescent female players in European football: a prospective study over one outdoor soccer season. Scand J Med Sci Sports.

[13] Soligard T, Myklebust G, Steffen K (2008). Comprehensive warm-up programme to prevent injuries in young female footballers: cluster randomised controlled trial. Br Med J.

[14] Backous DD, Friedl KE, Smith NJ, Parr TJ, Carpine WD Jr (1988). Soccer injuries and their relation to physical maturity. Am J Dis Child.

[15] Soligard T, Grindem H, Bahr R, Andersen TE (2010). Are skilled players at greater risk of injury in female youth football?. Br J Sports Med.

[16] Arnason A, Gudmundsson A, Dahl HA, Jóhannsson E (1996). Soccer injuries in Iceland. Scand J Med Sci Sports.

[17] Hawkins RD, Fuller CW (1999). A prospective epidemiological study of injuries in four English professional football clubs. Br J Sports Med.

[18] Hawkins RD, Hulse MA, Wilkinson C, Hodson A, Gibson M (2001). The association football medical research programme: an audit of injuries in professional football. Br J Sports Med.

[19] Gianotti M, Al-Sahab B, McFaull S, Tamim H (2011). Epidemiology of acute soccer injuries in Canadian children and youth. Pediatr Emerg Care.

[20] Steffen K, Emery CA, Romiti M (2013). High adherence to a neuromuscular injury prevention pro- gramme (FIFA 11+) improves functional balance and reduces injury risk in Canadian youth female football players: a cluster randomised trial. Br J Sports Med.

[21] American Academy of Pediatrics on Sports Medicine and American Academy of Orthopaedic Surgeons (2009). Care of the Young Athlete, 2nd Edition. Elk Grove Village, IL: American Academy of Pediatrics.

[22] Le Gall F, Carling C, Reilly T (2008). Injuries in young elite female soccer players: an 8-season prospective study. Am J Sports Med.

[23] Kirkendall DT, Marchak PM, Garrett WE (2002). A prospective 3-year injury incidence in youth soccer. Med Sci Sports Exerc.

[24] Collins CL, Fields SK, Comstock RD (2008). When the rules of the game are broken: what proportion of high school sports-related injuries are related to illegal activity?. Inj Prev.

[25] Ekstrand J, Gillquist J (1983). Soccer injuries and their mechanisms: a prospective study. Med Sci Sports Exerc.

[26] International Football Association Board Laws of the Game. Association Board.

[27] Shaw AD, Gustillo T, Court-Brown CM (1997). Epidemiology and outcome of tibial diaphyseal fractures in footballers. Injury.

[28] Templeton PA, Farrar MJ, Williams HR, Bruguera J, Smith RM (2000). Complications of tibial shaft soccer fractures. Injury.

[29] Leininger RE, Knox CL, Comstock RD (2007). Epidemiology of 1.6 million pediatric soccer related injuries presenting to United States emergency departments from 1990 to 2003. Am J Sports Med.

[30] Longo UG, Ciuffreda M, Locher J, Maffulli N, Denaro V (2016). Apophyseal injuries in children's and youth sports. Br Med Bull.

